# Aggravation of myocardial dysfunction by injurious mechanical ventilation in LPS-induced pneumonia in rats

**DOI:** 10.1186/1465-9921-14-92

**Published:** 2013-09-18

**Authors:** Lonneke Smeding, Jan Willem Kuiper, Frans B Plötz, Martin CJ Kneyber, AB Johan Groeneveld

**Affiliations:** 1 Institute for Cardiovascular Research ICaR-VU, VU University Medical Center, Amsterdam, Netherlands; 2 Department of Pediatric Intensive Care, VU University Medical Center, Amsterdam, Netherlands; 3 Department of Physiology, VU University Medical Center, Amsterdam, Netherlands; 4 Department of Cardiology, Academic Medical Center, Amsterdam, Netherlands; 5 Department of Pediatrics, Tergooi Hospital, Blaricum, Netherlands; 6 Department of Pediatrics, Division of Pediatric Intensive Care, Beatrix Children’s Hospital/University Medical Center Groningen, The University of Groningen, Groningen, Netherlands; 7 Critical Care, Anesthesiology, Peri-operative medicine and Emergency Medicine (CAPE), The University of Groningen, Groningen, Netherlands; 8 Department of Intensive Care, Erasmus University Medical Center, Rotterdam, Netherlands

**Keywords:** Biotrauma, Ventilator-induced lung injury, Myocardial depression, Endotoxin, Toll like receptor (TLR)2, Chemokine (C-X-C motif) ligand (CXCL)1, Heat shock protein (HSP)70

## Abstract

**Background:**

Mechanical ventilation (MV) may cause ventilator-induced lung injury (VILI) and may thereby contribute to fatal multiple organ failure. We tested the hypothesis that injurious MV of lipopolysaccharide (LPS) pre-injured lungs induces myocardial inflammation and further dysfunction *ex vivo,* through calcium (Ca^2+^)-dependent mechanism.

**Materials and methods:**

N = 35 male anesthetized and paralyzed male Wistar rats were randomized to intratracheal instillation of 2 mg/kg LPS or nothing and subsequent MV with lung-protective settings (low tidal volume (V_t_) of 6 mL/kg and 5 cmH_2_O positive end-expiratory pressure (PEEP)) or injurious ventilation (high V_t_ of 19 mL/kg and 1 cmH_2_O PEEP) for 4 hours. Myocardial function *ex vivo* was evaluated in a Langendorff setup and Ca^2+^ exposure. Key mediators were determined in lung and heart at the mRNA level.

**Results:**

Instillation of LPS and high V_t_ MV impaired gas exchange and, particularly when combined, increased pulmonary wet/dry ratio; heat shock protein (HSP)70 mRNA expression also increased by the interaction between LPS and high V_t_ MV. For the heart, C-X-C motif ligand (CXCL)1 and Toll-like receptor (TLR)2 mRNA expression increased, and ventricular (LV) systolic pressure, LV developed pressure, LV +dP/dt_max_ and contractile responses to increasing Ca^2+^ exposure *ex vivo* decreased by LPS. High V_t_ ventilation aggravated the effects of LPS on myocardial inflammation and dysfunction but not on Ca^2+^ responses.

**Conclusions:**

Injurious MV by high V_t_ aggravates the effects of intratracheal instillation of LPS on myocardial dysfunction, possibly through enhancing myocardial inflammation via pulmonary release of HSP70 stimulating cardiac TLR2, not involving Ca^2+^ handling and sensitivity.

## Introduction

Injurious mechanical ventilation (MV) can induce ventilator-induced lung injury (VILI) and may thereby contribute to multiple organ failure, potentially by spillover from lung-borne inflammatory mediators into the circulation which may act on distant organs such as the kidneys [1-3]. Direct or primary pulmonary injury, like pneumonia or aspiration, is associated with more severe VILI than indirect pulmonary injury, such as sepsis, in the presence of identical ventilatory settings [4].

In contrast to the kidney, the effect of VILI on the heart is less clear. Only a few studies have addressed the effect of MV on myocardial function, independent from a fall in venous return and cardiac performance following a rise in intrathoracic pressure [5]. It was, for instance, suggested that plasma from dogs ventilated with 15 cm H_2_O positive end expiratory pressure (PEEP) contains a negative inotropic mediator, which may adversely affect myocardial function [6]. Next, Nin et al. showed that high tidal volume (V_t_) ventilation induces VILI and upregulates myocardial cyclooxygenase (COX)-1 and COX-2 mRNA expression [7]. These enzymes are known to be upregulated during inflammatory conditions. Also, Brander et al. showed that high V_t_ ventilation during lung injury and associated VILI increased myocardial interleukin (IL) 8 expression, which was decreased with so-called lung-protective strategies [8]. We have shown that injurious MV (ie high tidal volume (V_t_) ventilation) for 4 hours augmented myocardial inflammation in a cecal ligation and puncture model through an enhanced deposition of advanced glycation end products resulting in increased myocardial inflammation [9]. These latter studies suggest that pulmonary overdistention and VILI may induce myocardial inflammation but the functional consequences were not examined.

Sepsis may hamper both pulmonary function [10], thereby necessitating MV, and myocardial function [11-14]. Sepsis may be associated with myocardial inflammation and a decrease in myocardial calcium (Ca^2+^) handling and sensitivity, thereby potentially contributing to impaired myocardial function [12,13]. Lung injury often results in (severe) hypoxia; as a consequence the function of the cardiomyocyte L-type Ca^2+^ current may be suppressed [15-17]. All of this suggests that VILI may cause myocardial dysfunction via a Ca^2+^ dependent pathway.

In the current study we used a two hit model of lipopolysaccharide (LPS)-induced pneumonia and subsequent high V_t_ ventilation to induce VILI and studied myocardial function *ex vivo.* Non-injurious low V_t_ ventilation served as control*.* We hypothesized that spillover of inflammatory mediators during VILI induces myocardial inflammation and decreases myocardial function through a Ca^2+^-dependent pathway.

## Materials and methods

### Animal experiments

All experiments applied with the Guide for Care and Use for laboratory animals of the National Institute of Health and were approved by the Institutional Animal Care and Use Committee of the VU University Amsterdam. Thirty-five male Wistar rats weighing 330 ± 20 g were anesthetized with 12.5 mg/kg midazolam (Pharmachemie BV, Haarlem, the Netherlands) and 85 mg/kg ketamine (Alfasan, Woerden, the Netherlands) i.p. and 10 mg/kg ketamine i.m. Anesthesia was maintained with 1.2 mg/kg/h midazolam and 20 mg/kg/h ketamine i.v. Paralysis was maintained with pancuronium 0.6 mg/kg/h i.v. to allow for high V_t_ ventilation. Animals were placed in supine position on a heating pad maintaining body temperature at 37°C. A tracheostomy was performed and a 14 Gauge canula was inserted into the trachea. During preparation, animals were ventilated with a V_t_ of 6 ml/kg and 5 cm H_2_O positive end-expiratory pressure (PEEP) (Avea, CareFusion, Houten, the Netherlands). Catheters were inserted into the left carotid artery and left jugular vein for arterial blood sampling and continuous measurement of the mean arterial pressure (MAP) and central venous pressure (CVP) in mmHg. Heart rate (HR) in beats per minute (BPM) was derived from MAP measurements. The femoral artery was catheterized with a thermistor from a pulmonary artery catheter to measure cardiac output. Cardiac output was obtained every 30 minutes by averaging two successive thermodilution determinations (CO Computer, 9520A, Edwards laboratory, Santa Ana, Ca, USA), for which 200 μl of cold saline was injected via the right jugular vein catheter as described previously [18]. Blood samples (150 μl) were taken every hour for gas analysis and these were replaced by equal volumes of normal saline. Blood gas analysis was performed using a pH blood-gas analyzer (ABL 50; Radiometer, Copenhagen, Denmark). Partial pressure of arterial oxygen pressure (P_a_O_2_, mmHg)/fraction of inspired oxygen (F_I_O_2_) ratios were calculated.

### Experimental protocol

After preparation, hemodynamics were allowed to stabilize for 10 min after which base line values were established (t=−10). At t=−5 animals were randomized to non-LPS treated or LPS treated groups. The latter received saline-dissolved LPS (2 mg/kg, LPS L2880, LPS from E. Coli 055:B5, Sigma-Aldrich) intratracheally using a miniature nebulizer (Penn-Century, Wyndmoor, PA, USA). Five minutes after LPS administration, the protocol was started and animals were randomly assigned to one of two ventilation strategies; ventilation with either low V_t_ (6 ml/kg, 5 cm H_2_O PEEP) which is regarded as a lung protective strategy [19], or high V_t_ (19 ml/kg, 1 cm H_2_O PEEP) which is regarded as an injurious strategy [20,21]. Ventilatory settings were chosen such that mean airway pressure was similar to avoid differences among groups in cardiac preloading [5,14]. Thus four groups were studied; non-LPS treated (n=8) and LPS-treated animals (n=8) ventilated with low V_t_ and non-LPS treated (n=11, including N = 3 animals not included in the Langendorff setup because of technical issues) and LPS-treated animals (n=8) ventilated with high V_t_. The F_I_O_2_ was set at 0.4 in all groups but was increased when oxygenation was impaired. Ventilation rate was set and adjusted if necessary throughout the experiment to maintain normocapnia. Four hours after the start of the protocol, hearts were rapidly dissected and myocardial function was measured *ex vivo*. If P_a_O_2/_F_I_O_2_ fell below 150 with a F_I_O_2_ of 100% and an increase in mean airway pressure of 2 cm H_2_O compared to t=0 was present, the experiment was terminated prematurely and myocardial function *ex vivo* was measured.

### Myocardial function ex vivo

Myocardial function *ex vivo* was measured in a Langendorff set-up (n=8 per group), to study myocardial function independent of loading condition as previously described [22]. Briefly, the aorta of the isolated heart was canulated and the heart was perfused with a modified Krebs-Henseleit solution at a constant coronary perfusion pressure of 80 mmHg at 37°C. The modified Krebs-Henseleit solution contained (in mM) 118.5 NaCl, 4.7 KCl, 25 NaHCO_3_, 1.2 MgCl_2_, 1.2 KH_2_PO_4_ and 11 glucose and was equilibrated with 95% O_2_ and 5% CO_2_ at a pH of 7.4. Solutions with different with CaCl_2_.2(H_2_O) concentrations were made, resulting in final Ca^2+^ concentrations of 0.5; 1; 2 and 4 mM. Both right and left atria were removed and hearts were paced at 5 Hz with electrodes. Afferent coronary flow was measured with a flow meter (Transonic Systems Europe B.V., Maastricht, the Netherlands). A custom-made balloon was inserted in the left ventricle to measure isovolumic pressures with a catheter tip manometer system and the heart was allowed to stabilize for 10 minutes. Ventricular volume at maximal pressure development (V_max_) was determined and balloons were adjusted to 85% of V_max_. Hearts were allowed to stabilize for 2 min. After stabilization, myocardial function was measured by left ventricular (LV) systolic and diastolic pressure, maximal rates of pressure development (+dP/dt_max)_ and pressure decline (−dP/dt_min_). As measurements were performed at a fixed heart rate and preload, +dP/dt_max_ and -dP/dt_min_ can be regarded as indices of contractility and relaxation, respectively [11]. Developed pressure was calculated as systolic pressure minus diastolic pressure. Force-pCa (i.e. –^10^log [Ca^2+^]) relations were fit to the Hill equation and subsequently the pCa_50,_ the pCa at which 50% of the developed force was reached, was calculated [23]. A decrease in the pCa_50_ indicates a decrease in Ca^2+^ sensitivity. After this protocol, hearts were removed from the isolated Langendorff-perfused heart set-up, cut transversely in three sections, frozen in liquid nitrogen and stored at −80°C. The apical section of the heart was used to calculate wet to dry weight ratio.

### Wet to dry weight ratios

Immediately after the animals were sacrificed, middle right lung lob was taken and weighed, dried at 37°C and weighed again. After freezing, apical section of the heart was weighed, freeze-dried and weighed again.

### mRNA expression

To study myocardial inflammatory response we randomly choose animals from each group in which we studied myocardial mRNA expression of the key pro-inflammatory mediators TNFα, interleukin (IL)6, IL1β, chemokine (C-X-C motif) ligand (CXCL)1 and TLR2. Myocardial tissue from the center transverse section and pulmonary tissue from the right lung were pulverized with a mortar and RNA was extracted using Trizol. Pulmonary RNA was purified using the RNA clean up kit (Qiagen, Venlo, the Netherlands). DNA was removed by DNAse I amplification grade (Invitrogen, Breda, The Netherlands) A total of 2 μg RNA was used to synthesize copy DNA (cDNA) using a Cloned AMV First Strand cDNA Synthesis Kit (Invitrogen, Breda, The Netherlands) using oligo-dT priming. Quantitive RT-PCR analysis was performed using SYBR Green in an ABI 7500 sequence detection system (Applied Biosystems, Foster City, USA). Briefly, 8 μl mix was prepared using 25 ng cDNA, forward and reverse primers and Mesa Green qPCR Mastermix Plus for SYBR assay (Eurogentec, Maastricht, The Netherlands). The used protocol was 2 min 50°C, 10 min 95°C, 40 cycles (0:15 min 95°C, 1:00 min 58°C) and a dissociation curve. Primer sequences are shown in Table 1. Cycle threshold values (Ct), the number of cycles required for the fluorescent signal to cross the threshold, were measured. Relative expression levels of target genes were calculated relative to the housekeeping gene β-actin with the formula 2^(Ct (β-actin)- Ct (target gene))^.

**Table 1:** Primer sequences used for RT-PCR

		
HSP70	Forward	AGGTGGATTAGAGGCTCTTT
	Reverse	AACCTAGGACTTGATTGCAGA
TNFα	Forward	ACAAGCCCGTAGCCCACGTC
	Reverse	AGGAGCACGTAGTCGGGGCA
IL6	Forward	GTCTCGAGCCCACCAGGAACG
	Reverse	AAGCCTCCGACTTGTGAAGTGGT
IL1β	Forward	GAGCCCGTCCTCTGTGACTCGT
	Reverse	AGGCCCAAGGCCACAGGGATT
CXCL1	Forward	TCGCCAATGAGCTGCGCTGT
	Reverse	CAAGGCAAGCCTCGCGACCAT
TLR2	Forward	GGGAAGGCCATTCTGCCCAGG
	Reverse	CGGAGGTTCACACAGGCTCGC
β-actin	Forward	GGCCAACCGTGAAAAGATGA
	Reverse	GGACAACACAGCCTGGATGG

#### Statistical analysis

To analyze the effects of LPS, V_t_ and their interaction, general estimated equations (GEE) were performed. With GEE effects of separate parameters as well as their interaction can be calculated, taking repeated measures over time in the same animals and baseline values as covariates into account when appropriate. A statistically significant interaction implies that the effect of high (vs low) V_t_ ventilation over time differs between LPS and non-LPS-treated animals. T-tests were performed to evaluate the effect of high V_t_ ventilation in LPS-treated animals on mRNA expression which were normally distributed. Spearman correlations were calculated for relations since parameters were distributed non-parametrically. Data are shown as mean ± SEM, exact P values are given if >0.001. A P-value < 0.05 was considered statistically significant.

## Results

Baseline characteristics were similar between the groups. Seven LPS-treated animals ventilated with high V_t_ died prematurely (4 after t=150 min; 1 after t=180 min; 2 after t=210 min).

### Hemodynamic measurements

**Figure 1:**
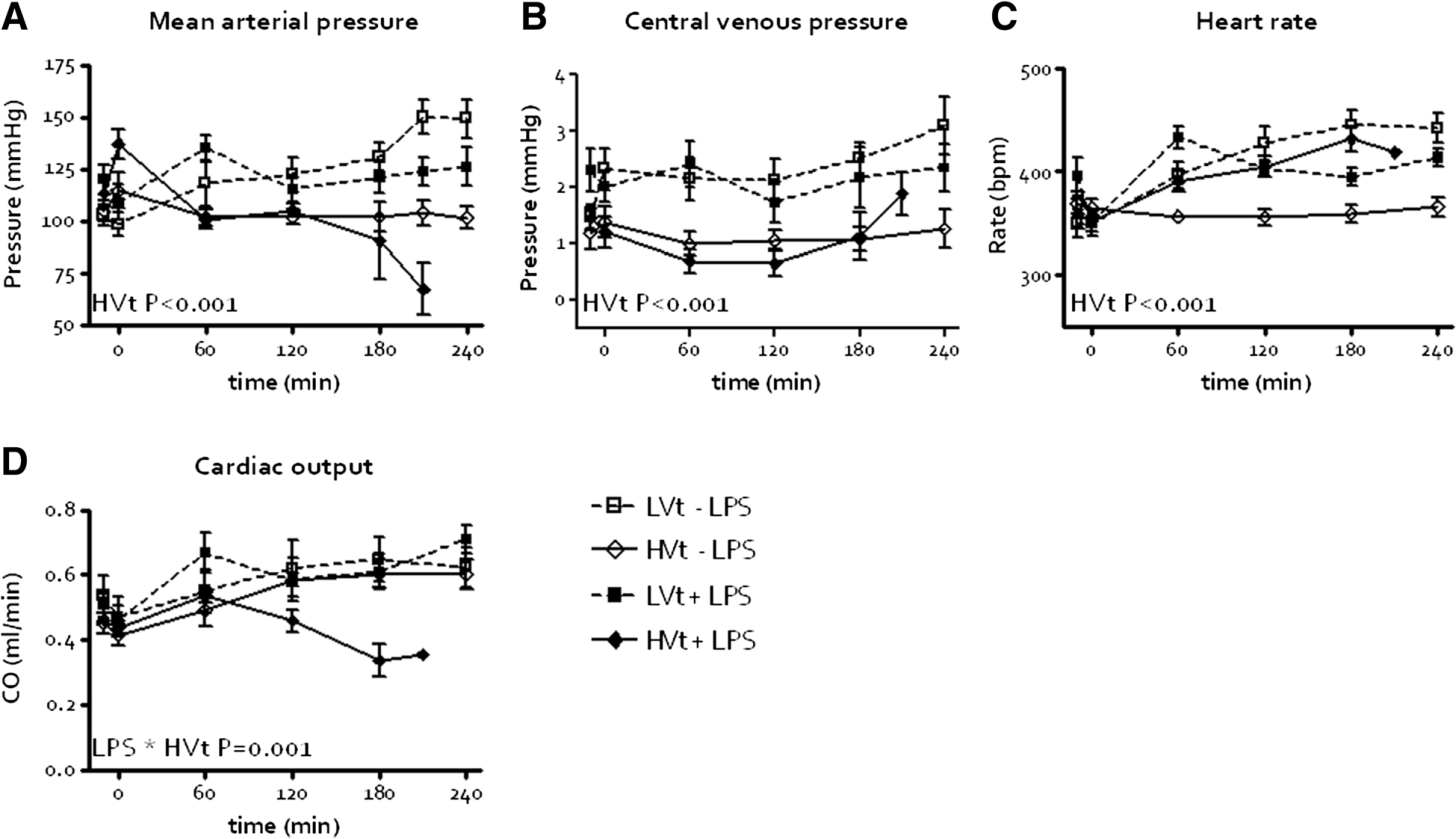
***In vivo *measurements in the course of time (LPS=lipopolysaccharide; LVt=low tidal volume (V**_**t**_**) ventilation, HVt=high V**_**t **_**ventilation).** Mean arterial pressure **(A)**, central venous pressure **(B)** and heart rate **(C)**. Cardiac output **(D)** decreased by the interaction between LPS installation and HVt ventilation.

**Figure 2:**
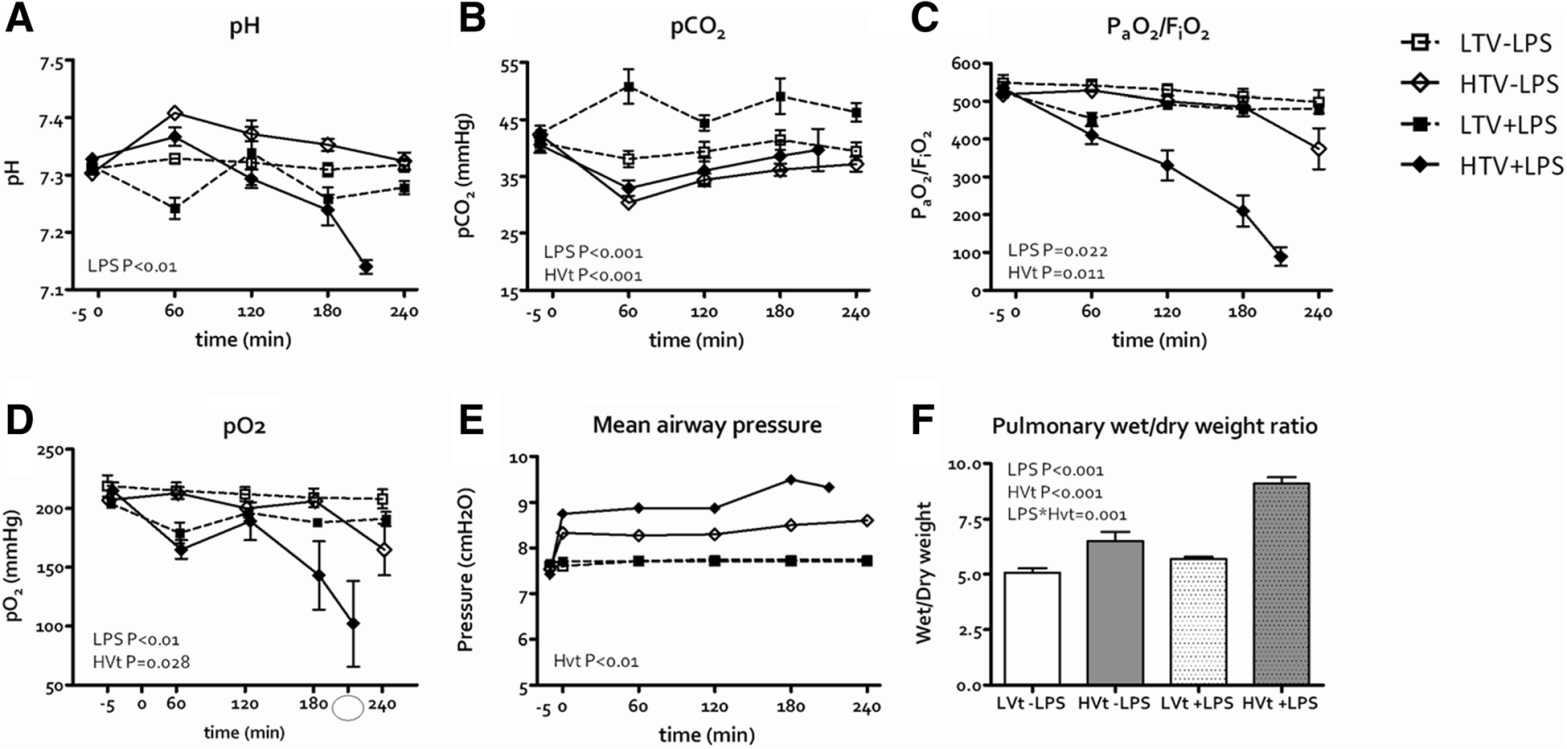
**Ventilator-induced lung injury induction in the course of time (LPS=lipopolysaccharide; LVt=low tidal volume (V**_**t**_**) ventilation, HVt=high V**_**t **_**ventilation). A**. pH. **B**. PCO_2_. **C**. P_a_O_2_/F_i_O_2_. **D**. PO_2_. **E**. Mean airway pressure. **F** Pulmonary wet/dry ratio increased by LPS and HVt ventilation interaction.

Hemodynamic data are shown in Figure 1. MAP (P<0.001), CVP (P<0.001) and HR (P<0.001) decreased by high V_t_ ventilation, but no effect of LPS was seen. Cardiac output decreased by the interaction between LPS and high V_t_ ventilation (P=0.001). Blood pH decreased by LPS (Figure 2A, P<0.001) but only LPS-treated animals ventilated with high V_t_ reached levels consistent with acidosis, a marker of hypoperfusion.

### Respiratory parameters

The P_a_CO_2_ increased by LPS (P<0.001) and decreased by high V_t_ ventilation (Figure 2B, P<0.001) but remained within the normal range in all cases. P_a_O_2_/F_I_O_2_ decreased by LPS (P=0.022) and high V_t_ ventilation (Figure 2C, P=0.011). The P_a_O_2_ decreased by LPS (P<0.01) and by high V_t_ ventilation (P = 0.028) but not by the interaction between LPS and high V_t_ ventilation (Figure 2D). In the high V_t_ ventilation + LPS group the lowest P_a_O_2_ was 62 mmHg at FiO_2_ 1.0. Mean airway pressure slightly increased by high V_t_ ventilation (Figure 2E, P<0.001) but was not affected by LPS. Pulmonary wet/dry weight ratio increased by both LPS (P<0.001) and high V_t_ ventilation (Figure 2F, P<0.001) and an interaction was seen (P=0.001).

### Myocardial function ex vivo

**Figure 3:**
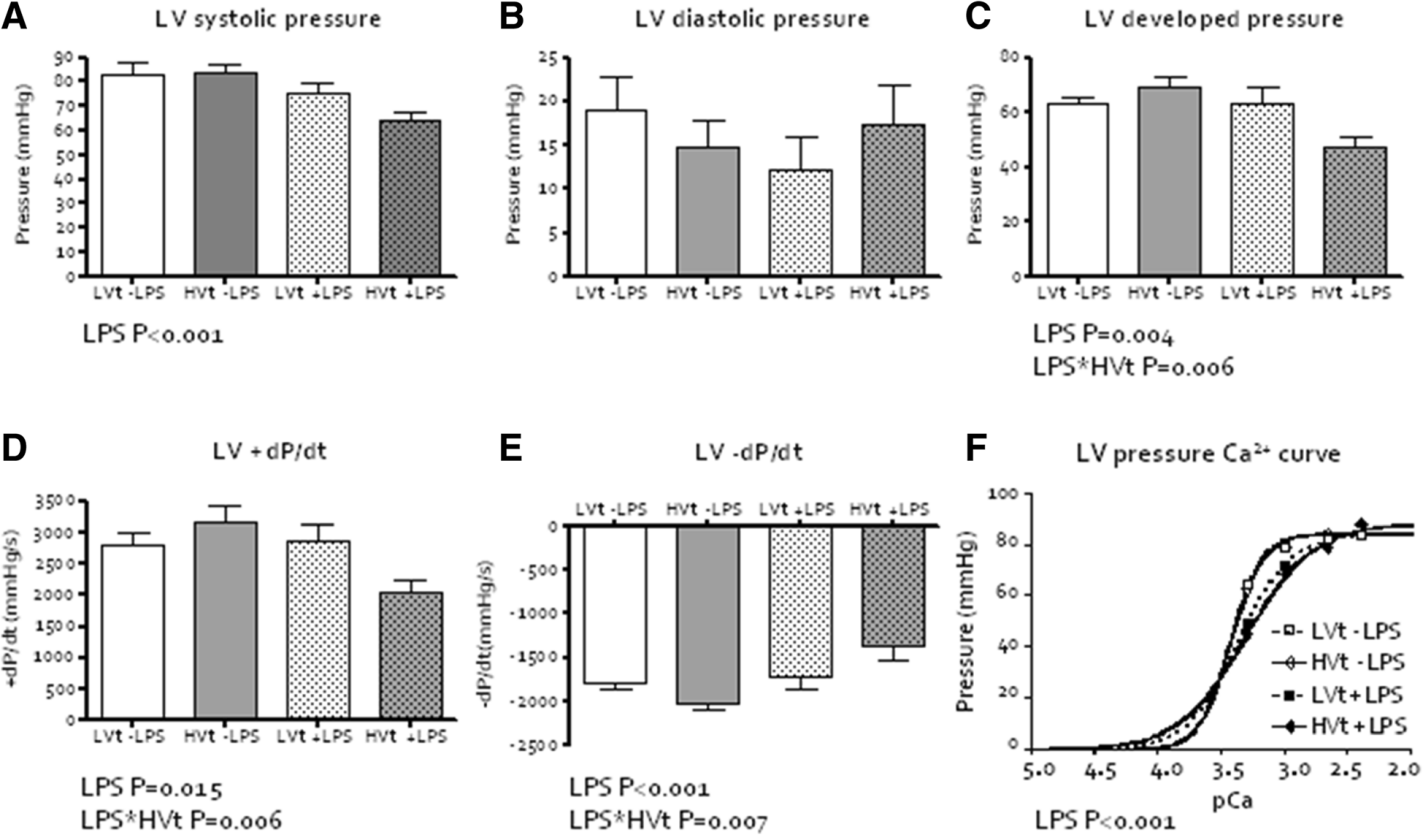
**Myocardial function *ex vivo *(LPS=lipopolysaccharide; LVt=low tidal volume (V**_**t**_**) ventilation, HVt=high V**_**t **_**ventilation). A**. Left ventricular (LV) systolic pressure. **B**. LV diastolic pressure. **C**. LV developed pressure decreased by the interaction between LPS installation and HVt ventilation. **D**. LV contractility, measured as +dP/_dtmax,_ decreased by the interaction between LPS installation and HVt ventilation. **E**. LV relaxation, measured as –dP/dt_min_, decreased by the interaction between LPS installation and HVt ventilation. **F**. LV pCa_50_.

LPS decreased myocardial contractile function *ex vivo* with a decrease in LV systolic pressure (Figure 3A, P<0.001), LV developed pressure (Figure 3C, P=0.004) and LV +dP/dt_max_ (Figure 3D, P=0.015). LPS also decreased relaxation with an increase in LV –dP/dt_min_ (Figure 3E, P=0.001). An interaction of LPS with high V_t_ ventilation was seen for LV developed pressure (P=0.006), LV +dP/dt_max_ (P=0.006) and LV -dP/dt_min_ (P=0.007), so that high V_t_ augmented the effect of LPS. Cardiac pCa_50_ decreased by LPS (Figure 3F, P<0.001) but was not affected by high V_t_ ventilation. Affluent coronary flow (16.0 ± 1.1 ml/min) and cardiac wet/dry weight ratio (6.6 ± 0.1) were similar between the groups excluding differences in perfusion or myocardial edema as contributing factors to dysfunction.

### Myocardial inflammation

Because we were unable to confirm the involvement of Ca^2+^ handling or sensitivity in our model, we searched for other explanations for the observed results. We therefore studied the well known pulmonary heat shock protein (HSP)70 response [24,25] and the myocardial toll-like receptor (TLR)2-induced pro-inflammatory response since the latter may be related [26]. Myocardial TNFα, IL6 and IL1β expressions were not affected by either LPS or high V_t_ ventilation. TLR2 mRNA expression increased by LPS (P=0.035) but not by high V_t_ ventilation. Myocardial CXCL1 mRNA expression increased by LPS (P=0.017), high V_t_ ventilation (P=0.004) and an interaction between LPS and high V_t_ was seen (P=0.013). CXCL1 (r=−0.60, P=0.002) and TLR2 (r=−0.54, P=0.008) mRNA expressions inversely correlated with LV systolic pressure across groups. TLR2 mRNA expression also correlated with LV –dP/dt_min_ (r=0.48, P=0.021). Pulmonary HSP70 mRNA expression increased by the interaction between LPS and high V_t_ ventilation (Table 2, P<0.001), whereas no effect on myocardial HSP70 mRNA expression was observed.

**Table 2:** Median values and interquartile ranges for pulmonary and myocardial mRNA expression in arbitrary units (A.U.)

	**LVt, LPS (−)**	**HVt, LPS (−)**	**LVt, LPS (+)**	**HVt, LPS (+)**
	**(N = 6)**	**(N = 6)**	**(N = 5)**	**(N = 6)**
Pulmonary mRNA expression (A.U.)				
**HSP70**	17*10^-3^	2.5*10^-3^	3.8*10^-3^	16*10^-3^#
	(8.7-26*10^-3^)	(0.050-8.2*10^-3^)	(3.4-6.2*10^-3^)	(8.4-27*10^-3^)
Myocardial mRNA expression (A.U.)				
**TLR2**	0.10*10^-3^	0.10*10^-3^	0.10*10^-3^	0.25*10^-3^
	(0–0.22*10^-3^)	(0–0.25*10^-3^)	(0.10-0.25*10^-3^)	(0.18-0.55*10^-3^)
**TNFα**	0.10*10^-3^	0.15*10^-3^	0.20*10^-3^	0.45*10^-3^
	(0–0.38*10^-3^)	(0.075-1.6*10^-3^)	(0.05-0.35*10^-3^)	(0.10-0.63*10^-3^)
**IL6**	0	0.05*10^-3^	0.10*10^-3^	0.35*10^-3^
	(0–0.35*10^-3^)	(0–3.1*10^-3^)	(0–7.0*10^-3^)	(0–4.7*10^-3^)
**IL1β**	0.45*10^-3^	1.3*10^-3^	1.6*10^-3^	1.9*10^-3^
	(0.075-3.9*10^-3^)	(0.38-15*10^-3^)	(0.65-2.6*10^-3^)	(0.83-4.9*10^-3^)
**CXCL1**	17*10^-3^	12*10^-3^	21*10^-3^	119*10^-3 ##^
	(2.7^3^-57.8*10^-3^)	(3.8-67*10^-3^)	(16-37*10^-3^)	(71-225*10^-3^)

Relative expression levels of target genes were calculated relative to the housekeeping gene β-actin with the formula 2^(Ct (β-actin)- Ct (target gene))^. *LPS* lipopolysaccharide, *LVt* low tidal volume (V_t_) ventilation, *HVt* high V_t_ ventilation, *HSP70* heat shock protein 70, *TLR2* toll like receptor 2, *TNF α* tumor necrosis factor α, *IL6* Interleukin 6, *IL1β* Interleukin 1β, *CXCL1* chemokine (C-X-C motif) ligand 1. The interaction between LPS and HVt ventilation increased pulmonary HSP70 (#, P<0.001) and myocardial CXCL1 mRNA expression (##, P=0.013).

## Discussion

In this study, we showed that injurious MV with high V_t_ in a model of LPS-induced pneumonia aggravated LPS-induced myocardial dysfunction *ex vivo*. In contrast with our hypothesis, we could not confirm alterations in Ca^2+^ sensitivity or calcium handling. Additional analyses suggested that myocardial dysfunction was potentially mediated through pulmonary release of HSP70 stimulating myocardial TLR2.

We used a two-hit model with intratracheal LPS installation and MV with V_t_ of 19 ml/kg to create VILI in line with previous studies [20,21]. This approach resulted in lung injury: pulmonary gas exchange deteriorated and the pulmonary wet-to-dry ratio increased, consistent with VILI. Mortality was high as in the LPS and high V_t_ group only one animal survived 4 hours, but this did not preclude *ex vivo* studies. Furthermore, myocardial function was impaired as demonstrated by a decreased CO *in vivo* without a fall in CVP, and a decreased LV systolic pressure, developed pressure and +dP/dtmax and increased –dP/dtmin *ex vivo* following LPS installation [5,21]. Moreover, we found that the LPS-induced myocardial dysfunction *in vivo* and *ex vivo* was aggravated by high V_t_ ventilation and development of VILI over time as shown by a further worsening of LV function parameters. It can be surmised that the mechanisms underlying VILI and myocardial dysfunction are comparable to those in sepsis, including myocardial inflammation [27]. In fact, MV may evoke an inflammatory response that is similar to that evoked by endotoxin, signifying a synergistic (i.e. double-hit) effect [28,29]. Injurious MV augments lung injury caused by intra-tracheal instillation of bacterial products [30,31]. Our results are in line with such a synergistic effect of MV in lungs and heart and we examined potential routes.

The causative mechanisms underlying the observed decrease in *ex vivo* myocardial function may be multifactorial. We hypothesized that high V_t_ ventilation caused myocardial dysfunction through a Ca^2+^ dependent manner. First, it can be postulated that distant organ failure after experimental injurious MV might be due to spillover of LPS from the injured lungs into the systemic circulation [31,32] which can then decrease myocardial function by inflammatory responses decreasing myocardial Ca^2+^ handling and sensitivity [12,13]. Although LPS-induced myocardial dysfunction in our study was associated with a decreased cardiac pCa_50_, high V_t_ ventilation did not affect cardiac pCa_50_, thereby excluding an effect on Ca^2+^ handling and sensitivity and rendering injurious MV-induced pulmonary release of LPS in the systemic circulation as a mediator unlikely. As we measured Ca^2+^ sensitivity in hearts with intact cell membranes, the Ca^2+^ sensitivity in this study should otherwise not be confused with myofilament sensitivity [23]. As we cannot distinguish between myofilament sensitivity and Ca^2+^ handling we cannot exclude the possibility that both parameters were altered. Second, acidosis may decrease myocardial function through a Ca^2+^-dependent mechanism [33]. Nonetheless, the acidosis we observed *in vivo* is less likely to be a cause of the observed dysfunction aggravated by injurious MV. Finally, hypoxia-induced cardiac dysfunction is also Ca^2+^-dependent as it effects the L-type Ca^2+^ channel [15-17]. However, we think that the level of hypoxia is also less likely to be cause of the observed dysfunction as there was no interaction between LPS and high V_t_ ventilation. Furthermore, inadequate delivery of O_2_ seems unlikely. In our experiments, hypoperfusion seems unlikely as a contributory factor because the coronary flow *ex vivo* was similar between groups. Myocardial edema may affect myocardial function but no difference in cardiac wet/dry weight ratio was found, thereby excluding edema as a causative factor [34].

Hence, we may conclude that the VILI-induced aggravation of myocardial dysfunction was most likely not mediated by a Ca^2+^-dependent pathway. In fact, our data suggest that a factor related to the severity of VILI contributed to myocardial inflammation and dysfunction, for instance via ventilator-induced lung-borne HSP70, was involved. Extracellular HSP70 can bind to myocardial TLR2 and may thereby cause an inflammatory response indicated by an increase in myocardial CXCL1 expression and Ca^2+^-independent myocardial dysfunction [26]. So, this route was subsequently studied when myocardial dysfunction appeared independent of Ca^2+^ handling in our experiments. We found that pulmonary but not myocardial HSP70 mRNA expression increased in severe VILI, after high V_t_ ventilation in combination with LPS exposure, as shown before [24,25]. We only may speculate about its involvement. In favor of our speculations is that it has been shown before that HSP70 may be actively released by cells under stress [35]. Indeed, injuring the lung with diesel exhaust, caused increases both pulmonary and systemic HSP70 expression [36]. Thus, HSPs may have a cytoprotective effect in VILI, but may also be involved in the activation of innate immunity when released into the systemic circulation. Explaining the high mRNA expression in the low V_t_ group in the absence of LPS is challenging as there is no explanation readily available. It has been discussed that the expression of HSP70 does not result from the transcription of a single gene, but is derived from what may be a complex interplay of several underlying genes [37]. It may thus be postulated that the HSP response following MV in the absence of LPS in our model is dependent upon both the degree of lung injury, the inflammatory response and their time course, so that we may have missed upregulation of HSP70 in the high V_t_ control group [38,39]. We have previously observed such a time course for TNF-α in a rat model of endotoxaemia-induced peritonitis [14]. This aspect warrants further investigation. Myocardial HSP70 mRNA expression was not increased, so that it is conceivable that pulmonary release of HSP70 had contributed to myocardial dysfunction. The myocardium of LPS-treated animals was probably more prone to bind lung-borne extracellular HSP70 by TLR2, since TLR2 was already upregulated by LPS in agreement with others [40]. We also found induction of CXCL1 after LPS and even more so after high V_t_ ventilation and CXCL1 mRNA expression inversely correlated with LV systolic pressure. Its exact role needs to be explored in further study. However, support for a Ca^2+^-independent role of CXC1 in myocardial dysfunction, as suggested by our study, comes from another report [26,41] and inhibition of CXCL1 decreases right ventricular failure in a model of pulmonary embolism [42]. In any case, myocardial expression of other inflammatory mediators was not increased, which might be due to a different time course of expression: TNF-α expression is increased 2 hours after LPS exposure, but not detectable anymore after 4 hours [14].

There are some limitations to the present study that may influence the interpretation of the main findings. First, mortality was high in the group of rats subjected to both high V_t_ ventilation and LPS. Only one animal completed the four-hour study protocol. This indicates that the time point at which the myocardial function was measured *ex vivo* as well as well when the organs were collected for analysis was not consistent among the animals in this group (the earliest being after 2.5 hrs of ventilation), but this does not invalidate our conclusions. Second, we were only able to measure mRNA expression levels and not the actual protein contents, but time may be needed for protein synthesis to become manifest after gene expression. Also, the concept that VILI-derived pulmonary HSP70 is directly responsible for myocardial inflammation and Ca^2+^-independent dysfunction warrants further investigation by mechanistic or intervention studies. We were unable to measure HSP70 in serum as its mechanistic role was not part of the primary hypothesis of our exploratory study. Our study was primarily designed as a proof of principle study to test the hypothesis that a Ca^2+^ dependent pathway was involved. When we refuted this hypothesis based on our findings, we explored alternative pathways in available tissue samples. Furthermore, measuring HSP70 in serum does not answer the question whether or not it is lung-borne, unless venous and arterial blood samples are taken and a pulmonary gradient can be measured.

In conclusion, our study shows that injurious ventilation with high V_t_ aggravates the effects of lung injury caused by intratracheal instillation of LPS on myocardial dysfunction, possibly through enhancing myocardial inflammation by lung-borne mediators, not involving Ca^2+^ handling and sensitivity.

## Competing interests

The authors declare that they have no competing interests.

## Authors’ contributions

LS designed the experimental set-up, performed the experiments and drafted and revised the manuscript. JWK advised in the experimental design and revised the manuscript. FBP, MCJK and ABJG conceived the study, designed the experimental set-up and revised the manuscript. All authors read and approved the final manuscript.
